# Preoperative Pectoralis Muscle Index Predicts Distant Metastasis-Free Survival in Breast Cancer Patients

**DOI:** 10.3389/fonc.2022.854137

**Published:** 2022-04-29

**Authors:** Wen-juan Huang, Meng-lin Zhang, Wen Wang, Qing-chun Jia, Jia-rui Yuan, Xin Zhang, Shuang Fu, Yu-xi Liu, Shi-di Miao, Rui-tao Wang

**Affiliations:** ^1^ Department of Internal Medicine, Harbin Medical University Cancer Hospital, Harbin Medical University, Harbin, China; ^2^ School of Computer Science and Technology, Harbin University of Science and Technology, Harbin, China

**Keywords:** breast cancer, skeletal muscle, distant metastasis, prognosis, overall survival

## Abstract

**Background:**

Breast cancer is one of the most commonly diagnosed cancers, and the fourth leading cause of cancer deaths in females worldwide. Sarcopenia is related to adverse clinical outcomes in patients with malignancies. Muscle index is a key parameter in evaluating sarcopenia. However, there is no data investigating the association between muscle index and distant metastasis in breast cancer. The aim of this study was to explore whether muscle index can effectively predict distant metastasis and death outcomes in breast cancer patients.

**Study Design:**

The clinical data of 493 breast cancer patients at the Harbin Medical University Cancer Hospital between January 2014 and December 2015 were retrospectively analyzed. Quantitative measurements of pectoralis muscle area and skeletal muscle area were performed at the level of the fourth thoracic vertebra (T4) and the eleventh thoracic vertebra (T11) of the chest computed tomography image, respectively. The pectoralis muscle index (PMI) and skeletal muscle index (SMI) were assessed by the normalized muscle area (area/the square of height). Survival analysis was performed using the log-rank test and Cox proportional hazards regression analysis.

**Result:**

The patients with metastases had lower PMI at T4 level (PMI/T4) and SMI at T11 level (SMI/T11) compared with the patients without metastases. Moreover, there were significant correlations between PMI/T4 and lymphovascular invasion, Ki67 expression, multifocal disease, and molecular subtype. In addition, multivariate analysis revealed that PMI/T4, not SMI/T11, was an independent prognostic factor for distant metastasis-free survival (DMFS) and overall survival (OS) in breast cancer patients.

**Conclusions:**

Low PMI/T4 is associated with worse DMFS and OS in breast cancer patients. Future prospective studies are needed.

## Introduction

Breast cancer is the most commonly diagnosed cancer, and the fourth leading cause of cancer-related death in women worldwide (1). Although many advancements have been made in treatment, some breast cancer patients continue to experience recurrence and metastasis. Therefore, it is of great significance to find new biomarkers for early prediction of metastasis.

Body composition including muscle mass and adipose tissue has been increasingly recognized as a key factor to predict long-term prognosis in various types of cancers such as breast cancer (2), colon cancer (3), and hepatocellular carcinoma (4). In the past few decades, body composition has mostly focused on evaluation of adipose tissue, because obesity and its clinical implications have been extensively investigated (5). Currently, muscle mass has become a focal point for clinical research (6). Sarcopenia refers to a generalized and progressive loss of skeletal muscle mass and function (7). Previous studies have proven that sarcopenia is related to adverse clinical outcomes of cancer, including disability, poor response to chemotherapy, post-operative complications, and reduced overall survival (OS) (2). Recent research found that sarcopenia was associated with survival in breast cancer (6). Cancer cachexia has long been recognized as a consequence of malignancy and occurs in approximately 40% of breast cancer patients, and is recognized as a direct cause of reduced quality of life (8). Differential diagnosis of cachexia and sarcopenia might be difficult in clinical practice because they share certain characteristics and overlap in some of the criteria (9). Cachexia is defined by international consensus as weight loss greater than 5%, or weight loss greater than 2% in individuals already showing depletion (10). Thus, accurately measuring muscle loss associated with sarcopenia may dramatically improve early detection of cachexia.

Computed tomography (CT) images, the preferred examination method, could evaluate skeletal muscle mass, adipose tissue amount and distribution, and tissue-specific radiodensity values (11). The cross-sectional area of skeletal muscle at the level of the third lumbar vertebra (L3) was directly correlated with whole-body skeletal muscle and fat mass in cancer patients (12). Reduced skeletal muscle radiodensity (SMD), referred to as myosteatosis, reflects intramuscular fat infiltration, and directly affects survival (13). Skeletal muscle index (SMI), a marker for muscle mass, and mean muscle attenuation (MA), a marker for muscle quality, are the independent negative predictors for OS in cancer patients. Some postmenopausal women with higher body fat levels have a higher risk of invasive breast cancer (5). However, recent studies revealed body composition plays different roles at different clinical stages of breast cancer (14). In early breast cancer, higher amount of visceral adipose tissue (VAT) and the lower quality of VAT represented by the Hounsfield unit (HU) were associated with shorter distant disease-free survival (15).

Conventionally, CT-measured skeletal muscle mass is usually evaluated at the level of the L3 (16). However, in patients with respiratory or breast disease, abdominal CT scans are not routinely performed for evaluation and follow-up. A chest CT scan is a common examination to evaluate breast cancer. Recent studies have observed that pectoralis muscle area (PMA), measured from CT scans of the chest, is associated with prognosis in lung cancer (17) and diffuse large B-cell lymphoma (18). Currently, the measurable indicators of muscle mass at the T4 level include skeletal muscle area (SMA) and PMA (19), but SMA in the upper thorax varies depending on upper limb positions (i.e., arms above the head versus arms by the side) (20). Therefore, PMA on chest CT imaging was used to assess the muscle area in our study.

The purpose of this research was to determine whether preoperative PMA on chest CT imaging could predict distant metastasis and OS in breast cancer patients.

## Patients and Methods

### Study Population

493 consecutive patients who underwent complete surgical resection for breast cancer at Harbin Medical University Cancer Hospital from January 1, 2014 to December 31, 2015 were included in this analysis. None of the patients received any therapy before the operation. The inclusion criteria for this study were as follows: (1) female patients at diagnosis were no less than 18 years old; (2) all patients were confirmed diagnosed with breast cancer by pathology; and (3) the patients had complete clinical and follow-up data. Exclusion criteria included: a history of malignancy, metastatic disease at diagnosis, neoadjuvant chemotherapy, incomplete clinical data and chest CT images, and loss to follow-up. Distant metastasis was defined as disease recurrence in distant organs and/or tissues that did not constitute local recurrence or regional recurrence, and was confirmed with imaging studies or pathologic examination of tissue samples. Information on follow-up and distant metastasis was obtained from each patient’s medical and imaging records (21). Distant metastasis-free survival (DMFS) was calculated from the date of surgery to the date of distant metastasis or the last follow-up. OS was defined as the time from the date of surgery to the date of either death or last follow-up. Patients were followed up every 3 months for the first 2 years, and every 6 months for years 3-5 after the operation. The final follow-up was completed on December 31, 2020. The median follow-up time was 65 months.

The study was approved by the Institutional Ethics Review Board of Harbin Medical University Cancer Hospital. Because of the retrospective nature of the research, the requirement for informed consent was waived.

### Data Collection

The following clinical variables were collected, including age at baseline, menstrual status, tumor size, lymph node metastasis, histopathological type, lymphovascular invasion, proliferation index (Ki67) expression, estrogen receptor (ER) status, progesterone receptor (PR) status, molecular classification, clinical stage, and postoperative treatment. Human epidermal growth factor receptor 2 (HER2) positivity was defined as immunohistochemistry (IHC) 3+ or fluorescence *in situ* hybridization (FISH) positive of the primary tumor.

### Body Composition Analysis

BMI (kg/m^2^) was calculated by dividing weight (kg) with height squared (m^2^). Cross-sectional chest CT images were used to assess body composition mass: SMA (cm^2^), PMA (cm^2^), visceral fat tissue area (VFA, cm^2^), and subcutaneous fat tissue area (SFA, cm^2^) were determined by using Image J software version 1.53a (Wayne Rasband, National Institutes of Health, USA). The software can segment tissue boundaries based on CT HU. According to previous studies, the HU thresholds were set from − 29 to + 150 for the skeletal muscles, − 150 to − 50 for visceral fat tissues, and − 190 to − 30 for subcutaneous fat tissues (22, 23). The areas (cm^2^) were divided by the square of height (m^2^) to obtain the SMI (cm^2^/m^2^), pectoralis muscle index (PMI, cm^2^/m^2^), visceral fat index (VFI, cm^2^/m^2^), and subcutaneous fat index (SFI, cm^2^/m^2^) (4, 16). Because there were no CT images available on level L3 for the breast cancer patients, CT images on the level of the fourth thoracic vertebra (T4) and the eleventh thoracic vertebra (T11) were used as alternatives for the assessment of the SMA. Skeletal muscles include all muscles at the T11 level. The pectoralis major and pectoralis minor muscle areas that were segmented bilaterally were used to evaluate PMA at the T4 level (18, 24). Areas that were not obviously targeted by muscle or fat tissues were deleted by manual manipulation. The CT images were analyzed by two independent readers who were blinded to clinical data, and the mean of the two measurements was used. The intra-observer coefficient of variation was less than 1.2%. The CT scans were obtained within 2 weeks before surgery, and the investigators who performed the measurements were blinded to the postoperative outcomes.

### Statistical Analysis

The descriptive statistics were presented as means ± SD or medians (interquartile range) for continuous variables, and percentages of the number for categorical variables. The Student’s t-test or the Mann-Whitney U test was used to determine the difference between the two groups. The Chi-square test was used for categorical variables between groups. To compare the differences in body composition parameters and find the potential factors for DMFS, the breast cancer patients were divided into two groups according to the later development of metastatic disease. The optimal cut-off value of PMI was calculated by the receiver operating characteristic (ROC) curve. Estimates of DMFS and OS were from Kaplan-Meier curves and tests of differences by log-rank test. Univariate and multivariate analyses were performed to identify the independent prognostic factors for DMFS and OS. The variables with P < 0.10 in the univariate Cox regression analysis were further analyzed using multivariate Cox regression. All statistical tests were conducted using SPSS version 25.0 software (SPSS Inc., Chicago, IL, USA). A two-tailed P value less than 0.05 was considered statistically significant.

## Results

This study involved 493 breast cancer patients. Among them, the mean age was 50.4 ± 8.7 years (range 26-79). There were 238 (48.3%) pre-menopausal and 255 (51.7%) post-menopausal patients. Most patients were diagnosed with tumor grade I/II disease, and the most common histological type was ductal cancer. The numbers of patients with Luminal-A, Luminal-B, HER2+, and TNBC were 151, 139, 103, and 100, respectively.

The clinical and pathological characteristics of breast cancer patients according to metastasis status are presented in **Table 1**. The patients with metastases were older and had lower PMA/T4, PMI/T4, SMA/T11, and SMI/T11 levels compared with the patients without metastases. There were significant differences in menopausal status, tumor size, Ki67 expression, ER status, PR status, molecular subtype, and adjuvant hormonal therapy between the two groups. However, BMI, histopathological type, multifocal disease, lymphovascular invasion, HER2 status, clinical stage, adjuvant radiotherapy, and adjuvant chemotherapy had no difference between the two groups.

**Table 1:** Baseline characteristics of breast cancer patients according to later development of metastatic disease.

Variables	Without metastases	With metastases	*P *value
N	447	46	
Age (years)	50.1 ± 8.5	53.4 ± 10.2	0.015
BMI (kg/m^2^)	24.1 ± 3.2	24.0 ± 3.1	0.850
PMA/T4 (cm^2^)	38.7 ± 12.4	32.8 ± 10.7	0.002
PMI/T4 (cm^2^/m^2^)	23.2 ± 14.9	13.1 ± 4.5	<0.001
SMA/T11 (cm^2^)	66.9 ± 19.6	55.7 ± 14.0	<0.001
SMI/T11 (cm^2^/m^2^)	25.7 ± 7.6	22.1 ± 5.8	<0.001
SFA/T11 (cm^2^)	162.1 ± 60.5	145.0 ± 54.7	0.066
SFI/T11 (cm^2^/m^2^)	49.3 ± 26.1	41.7 ± 24.2	0.058
VFA/T11 (cm^2^)	71.9 ± 46.7	58.9 ± 37.8	0.068
VFI/T11 (cm^2^/m^2^)	27.8 ± 18.3	23.3 ± 14.9	0.107
Menopausal status			0.011
Pre	224 (50.1)	14 (30.4)	
Post	223 (49.9)	32 (69.6)	
Histologic type			0.400
Ductal	425 (95.1)	45 (97.8)	
Others	22 (4.9)	1 (2.2)	
Multifocal disease			0.402
Yes	58 (13.0)	8 (17.4)	
No	389 (87.0)	38 (82.6)	
Tumor size (cm)			0.004
≥ 2.5	157 (35.1)	26 (56.5)	
< 2.5	290 (64.9)	20 (43.5)	
Lymph node status			0.062
Negative	220 (49.2)	16 (34.8)	
Positive	227 (50.8)	30 (65.2)	
Clinical stage			0.869
I-II	383 (85.7)	39 (84.8)	
III	64 (14.3)	7 (15.2)	
Lymphovascular invasion			0.199
Yes	152 (34.0)	20 (43.5)	
No	295 (66.0)	26 (56.5)	
Ki-67 (%)			<0.001
< 20%	132 (29.5)	1 (2.2)	
≥ 20%	315 (70.5)	45 (97.8)	
ER			0.001
Positive	316 (70.7)	21 (45.7)	
Negative	131 (29.3)	25 (54.3)	
PR			<0.001
Positive	292 (65.3)	15 (32.6)	
Negative	155 (34.7)	31 (67.4)	
HER2 status			0.988
Positive	155 (34.7)	16 (34.8)	
Negative	292 (65.3)	30 (65.2)	
Molecular subtype			<0.001
Luminal-A	150 (33.6)	1 (2.2)	
Luminal-B	123 (27.5)	16 (34.8)	
HER2-enriched	89 (19.9)	14 (30.4)	
TNBC	85 (19.0)	15 (32.6)	
Adjuvant radiotherapy			0.575
Yes	114 (25.5)	10 (21.7)	
No	333 (74.5)	36 (78.3)	
Adjuvant hormonal therapy			0.001
Yes	223 (49.9)	11 (23.9)	
No	224 (50.1)	35 (76.1)	
Adjuvant chemotherapy			0.855
Yes	430 (96.2)	44 (95.7)	
No	17 (3.8)	2 (4.3)	

BMI, body mass index; SMA, skeletal muscle area; SMI, skeletal muscle index; SFA, subcutaneous fat tissue area; SFI, subcutaneous fat index; VFA, visceral fat tissue area; VFI, visceral fat index; PMA, pectoralis muscle area; PMI, pectoralis muscle index; ER, estrogen receptor; PR, progesterone receptor; HER2, human epidermal growth factor receptor 2; TNBC, triple negative breast cancer; T4, fourth thoracic vertebra; T11, eleventh thoracic vertebra. P-value was obtained using Chi-square test.

**Figure 1:**
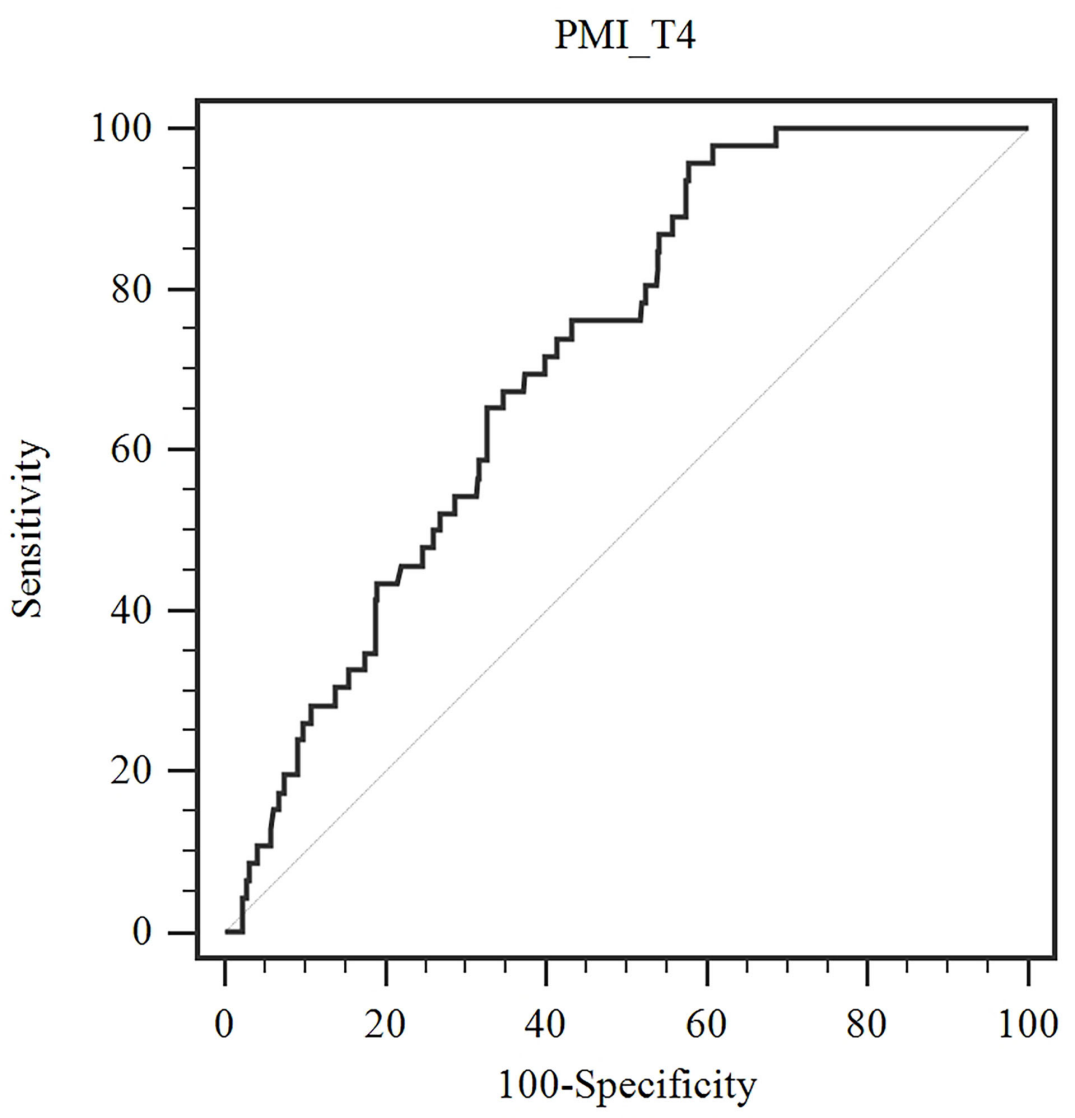
Optimized cut-off value was determined for PMI/T4 using standard ROC curve analysis.

Next, we identified the optimal cut-off value of PMI/T4 for distant metastasis by ROC under the curve analysis. According to the ROC curve analysis, the optimal cut-off value for PMI/T4 was 19.5 (cm^2^/m^2^), with sensitivity of 95.7%, and specificity of 42.3% (AUC = 0.715, 95% CI: 0.673-0.755, p < 0.0001) (**Figure 1**). The patients were divided into two risk stratification groups. If patients had low PMI/T4, they were classified into the high-risk group (n = 302). Conversely, if patients had high PMI/T4, they were classified into the low-risk group (n = 191). The relationships between PMI/T4 levels and clinical characteristics are summarized in **Table 2**. Our results showed that PMI/T4 was associated with BMI, lymphovascular invasion, Ki67 expression, multifocal disease, molecular subtype, and adjuvant chemotherapy. However, the associations between age, menopausal status, histopathological type, tumor size, lymph node status, clinical stage, ER status, PR status, HER2 status, adjuvant radiotherapy, adjuvant hormonal therapy, and PMI/T4 were not statistically significant.

**Table 2:** Baseline clinico-pathological parameters of breast cancer patients according to PMI/T4 levels.

Variables	>19.5 cm^2^/m^2^	≤19.5 cm^2^/m^2^	*P* value
N	191	302	
Age (years)	49.9 ± 8.3	50.8 ± 9.0	0.276
BMI (kg/m^2^)	24.6 ± 3.4	23.9 ± 3.1	0.022
Menopausal status			0.823
Pre	91 (47.6)	147 (48.7)	
Post	100 (52.4)	155 (51.3)	
Histologic type			0.402
Ductal	184 (96.3)	286 (94.7)	
Others	7 (3.7)	16 (5.3)	
Multifocal disease			<0.001
Yes	9 (4.7)	57 (18.9)	
No	182 (95.3)	245 (81.1)	
Tumor size (cm)			0.985
≥ 2.5	71 (37.2)	112 (37.1)	
< 2.5	120 (62.8)	190 (62.9)	
Lymph node status			0.077
Negative	101 (52.9)	135 (44.7)	
Positive	90 (47.1)	167 (55.3)	
Clinical stage			0.509
I-II	166 (86.9)	256 (84.8)	
III	25 (13.1)	46 (15.2)	
Lymphovascular invasion			<0.001
Yes	47 (24.6)	125 (41.4)	
No	144 (75.4)	177 (58.6)	
Ki-67 (%)			0.003
< 20%	66 (34.6)	67 (22.2)	
≥ 20%	125 (65.4)	235 (77.8)	
ER			0.139
Positive	138 (72.3)	199 (65.9)	
Negative	53 (27.7)	103 (34.1)	
PR			0.124
Positive	127 (66.5)	180 (59.6)	
Negative	64 (33.5)	122 (40.4)	
HER2 status			0.409
Positive	62 (32.5)	109 (36.1)	
Negative	129 (67.5)	193 (63.9)	
Molecular subtype			<0.001
Luminal-A	70 (36.6)	81 (26.8)	
Luminal-B	47 (24.6)	92 (30.5)	
HER2-enriched	34 (17.8)	69 (22.8)	
TNBC	40 (20.9)	60 (19.9)	
Adjuvant radiotherapy			0.528
Yes	51 (26.7)	73 (24.2)	
No	140 (73.3)	229 (75.8)	
Adjuvant hormonal therapy			0.389
Yes	86 (45.0)	148 (49.0)	
No	105 (55.0)	154 (51.0)	
Adjuvant chemotherapy			0.026
Yes	179 (93.7)	295 (97.7)	
No	12 (6.3)	7 (2.3)	

BMI, body mass index; SMI, skeletal muscle index; ER, estrogen receptor; PR, progesterone receptor; HER2, human epidermal growth factor receptor 2; TNBC, triple negative breast cancer; T4, fourth thoracic vertebra. P-value was obtained using Chi-square test.

**Figure 2:**
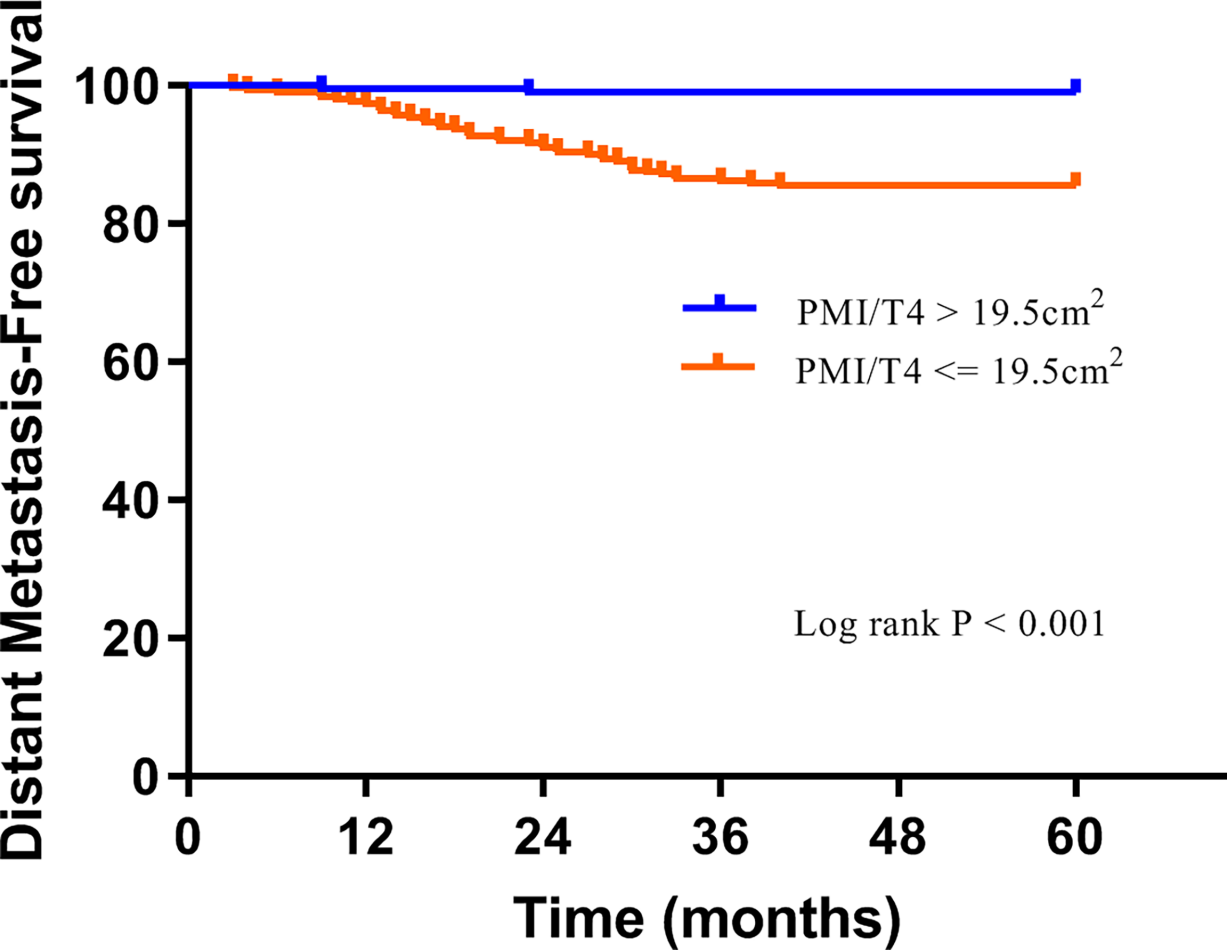
Distant metastasis-free survival curves of subgroups divided by high PMI/T4 and low PMI/T4.

**Figure 3:**
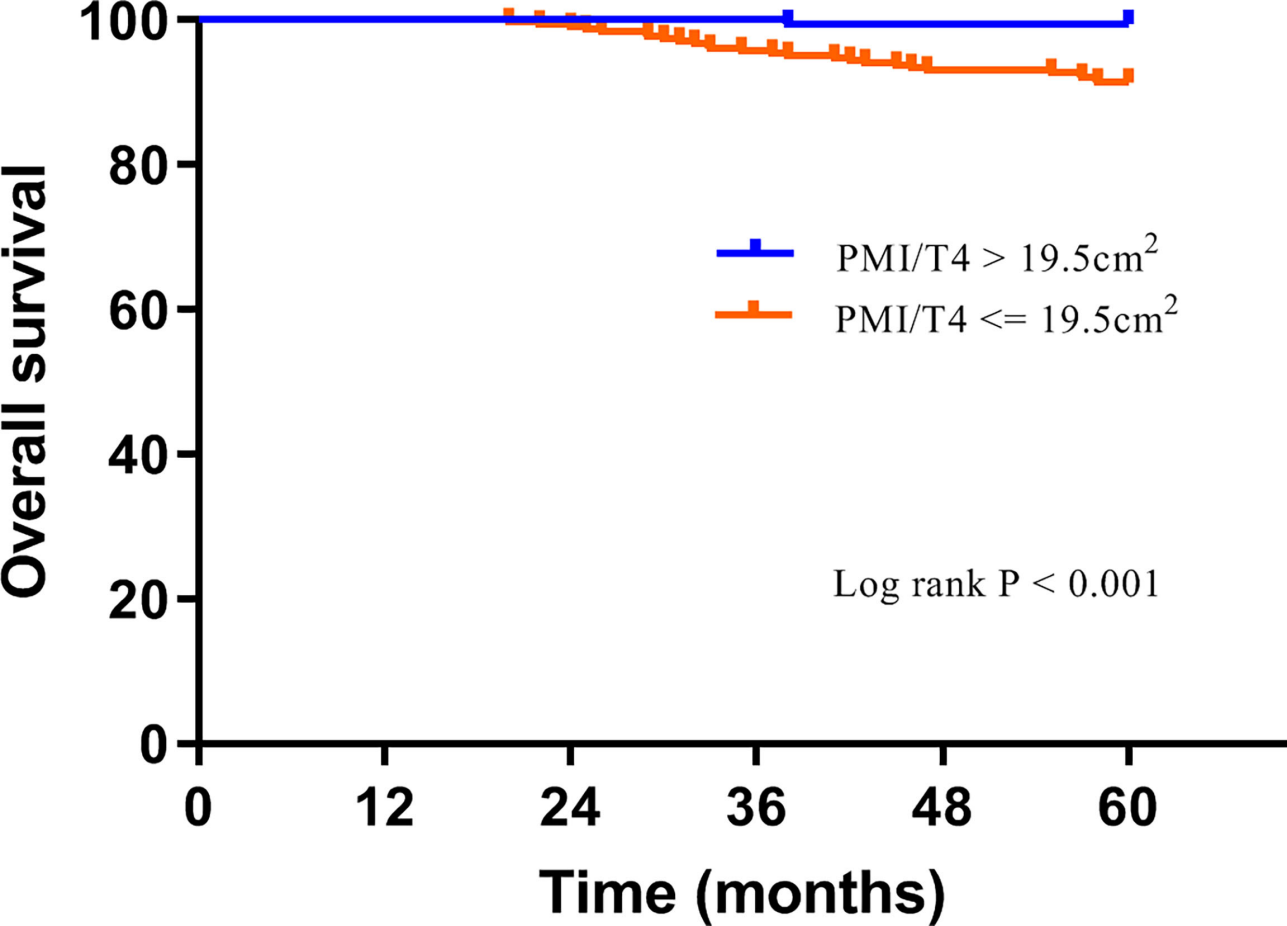
Overall survival curves of subgroups divided by high PMI/T4 and low PMI/T4.

The median follow-up was 65 months (interquartile range, 62-69 months), with 5-year DMFS and OS being 90.7% and 94.5%, respectively. Overall, 46 (9.3%) cases had distant metastasis, and 27 (5.5%) patients died during follow-up. The Kaplan-Meier analysis was applied by log-rank test to assess the prediction capacity of PMI/T4. Patients with high PMI/T4 had better DMFS and OS than those with low PMI/T4 (log-rank P < 0.001) (**Figures 2**, **3**).

Univariate and multivariate Cox regression analyses were performed in **Tables 3**, **4**. Univariate analyses revealed that age of diagnosis, menopausal status, Ki67 expression, tumor size, molecular subtype, PMI/T4, and adjuvant hormonal therapy had a significant correlation with DMFS. Ki67 expression, lymph node status, molecular subtype, PMI/T4, SMI/T11, and adjuvant hormonal therapy were significantly associated with OS. All the parameters in the univariate analysis with a p-value less than 0.10 were then incorporated into a multivariate Cox proportional hazards regression model. Multivariate Cox analysis confirmed that Ki67 expression and PMI/T4 were the independent prognostic variables for DMFS. Lymph node status and PMI/T4 were the independent prognostic factors for OS.

**Table 3:** The predictors of distant metastases in patients with breast cancer.

Variable	Univariate analysis		Multivariate analysis	
	HR (95% CI)	P-value	HR (95% CI)	P-value
Age (years)	1.043 (1.009–1.078)	0.012	1.035 (0.992–1.081)	0.114
BMI (kg/m^2^)	0.991 (0.936–1.050)	0.766		
Menopausal status				
(Post vs Pre)	2.210 (1.180–4.142)	0.013	1.251 (0.577–2.710)	0.571
Ki-67 (%)				
(≥ 20 vs < 20)	17.570 (2.422–127.464)	0.005	12.242 (1.667–89.890)	0.014
Histologic type				
(Ductal vs Others)	0.434 (0.060–3.147)	0.409		
Multifocal disease				
(Positive vs Negative)	1.385 (0.656–2.925)	0.393		
Tumor size (cm)				
(≥ 2.5 vs < 2.5)	2.272 (1.268–4.070)	0.006	1.702 (0.938–3.807)	0.080
Lymph node status				
(Positive vs Negative)	1.764 (0.961–3.235)	0.067	1.245 (0.666–2.327)	0.492
Clinical stage				
(III vs I-II)	1.065 (0.477–2.382)	0.877		
Lymphovascular invasion				
(Yes vs No)	1.446 (0.807–2.590)	0.215		
Molecular subtype	1.458 (1.045–2.034)	0.027	1.373 (0.975–1.935)	0.070
Adjuvant chemotherapy				
(Yes vs No)	0.839 (0.203–3.462)	0.808		
Adjuvant radiotherapy				
(Yes vs No)	0.836 (0.415–1.685)	0.616		
Adjuvant hormonal therapy				
(Yes vs No)	0.332 (0.168–0.653)	0.001	0.593 (0.258–1.363)	0.218
PMI/T4 (cm^2^/m^2^)	0.904 (0.861–0.949)	0.002	0.927 (0.886–0.969)	0.001
SMI/T11 (cm^2^/m^2^)	0.928 (0.885–0.973)	0.067	0.999 (0.944–1.057)	0.973

BMI, body mass index; SMI, skeletal muscle index; PMI, pectoralis muscle index; T4, fourth thoracic vertebra; T11, eleventh thoracic vertebra; HR, hazard ratio; CI, confidence interval.

**Table 4:** The predictors of OS in patients with breast cancer.

Variable	Univariate analysis		Multivariate analysis	
	HR (95% CI)	P-value	HR (95% CI)	P-value
Age (years)	1.026 (0.983–1.071)	0.243		
BMI (kg/m2)	0.985 (0.875–1.109)	0.803		
Menopausal status				
(Post vs Pre)	1.897 (0.852–4.222)	0.117		
Ki-67 (%)				
(≥ 20 vs < 20)	9.941 (1.349–73.258)	0.024	6.956 (0.935–51.779)	0.058
Histologic type				
(Ductal vs Others)	0.046 (0.000–117.322)	0.442		
Multifocal disease				
(Positive vs Negative)	1.115 (0.386–3.225)	0.840		
Tumor size (cm)				
(≥ 2.5 vs < 2.5)	1.353 (0.633–2.890)	0.435		
Lymph node status				
(Positive vs Negative)	3.325 (1.342–8.238)	0.009	2.879 (1.026–8.077)	0.045
Clinical stage				
(III vs I-II)	1.364 (0.516–3.601)	0.531		
Lymphovascular invasion				
(Yes vs No)	2.043 (0.960–4.347)	0.064	0.961 (0.406–2.274)	0.927
Molecular subtype	1.794 (1.253–2.568)	0.001	1.549 (0.969–2.477)	0.068
Adjuvant chemotherapy				
(Yes vs No)	1.067 (0.145–7.863)	0.949		
Adjuvant radiotherapy				
(Yes vs No)	1.050 (0.444–2.483)	0.911		
Adjuvant hormonal therapy				
(Yes vs No)	0.377 (0.160–0.893)	0.027	0.818 (0.275–2.430)	0.718
PMI/T4 (cm2/m2)	0.914 (0.861–0.970)	0.003	0.932 (0.874–0.993)	0.029
SMI/T11 (cm2/m2)	0.920 (0.864–0.980)	0.009	0.974 (0.905–1.048)	0.483

BMI, body mass index; SMI, skeletal muscle index; PMI, pectoralis muscle index; T4, fourth thoracic vertebra; T11, eleventh thoracic vertebra; HR, hazard ratio; CI, confidence interval.

## Discussion

This study showed that PMI/T4 was significantly associated with lymphovascular invasion, Ki67 expression, multifocal disease, and molecular subtype. In addition, PMI/T4 was an independent prognostic factor for DMFS and OS.

Sarcopenia, a useful parameter for reflecting body composition, was associated with the survival of breast cancer patients (6). Our results are consistent with those of previous studies. A previous report demonstrated that sarcopenia (defined as SMI/L3<41 cm^2^/m^2^) was an independent prognostic factor for disease-free survival (DFS) and OS in early breast cancer (25). Furthermore, another large study found that non-metastatic breast cancer patients with sarcopenia (defined as SMI/L3<40 cm^2^/m^2^) had higher overall mortality compared with those without (26). However, those studies just highlighted that sarcopenia is significantly associated with DFS and OS in patients with breast cancer (25, 26). To our knowledge, our study is the first to explore whether PMI can effectively predict DMFS in BC patients. DMFS is of vital clinical significance since distant metastasis is associated with a high rate of mortality and a difference in prognoses and responses to therapy in breast cancer patients (27). In addition, a close relationship between sarcopenia and metastasis has been verified in various types of cancers, including lung cancer (28), nasopharyngeal carcinoma (29), and colorectal cancer (30).

The mechanisms by which sarcopenia confers an increased risk of distant metastasis and mortality are still unclear, but the following reasons can be hypothesized. First, cancer cells utilize glucose and glutamine as a carbon skeleton and produce energy through lactate fermentation (31). It has been reported that high mobility group box-1 protein (HMGB1) secreted during tumorigenesis induces the degradation of host muscle tissues to supply glutamine to cancer cells as an energy source (32). Thus, expenditure of glutamine would lead to loss of muscle mass (33). Second, some studies found that myokines secreted from muscle cells could inhibit cancer cell growth and migration, and induce cancer cell death (34, 35). Therefore, we speculate that cancer cells utilize skeletal muscle as an energy repository, causing sarcopenia. Moreover, loss of muscle mass can lead to an impaired myokine response and an increased risk of distant metastasis and mortality.

Interestingly, our study revealed that PMI/T4, not SMI/T11, was an independent prognostic factor for DMFS and OS in breast cancer patients. Only two studies have been published on the association between PMA and breast cancer. One study demonstrated a close correlation between PMA on MRI and the psoas muscle area on CT scan in breast cancer patients (36). Another study showed that PMA on MRI is reduced after neoadjuvant chemotherapy (37). However, there is no study investigating the clinical implications of the PMI/T4 in CT images on distant metastasis and death outcome for breast cancer patients. A previous study found that low PMI/T4 was a risk factor for worse OS in lung cancer patients (17). Another study also detected that low PMI/T4 was strongly associated with worse PFS and OS in patients with diffuse large B-cell lymphoma (18). Our results confirmed that PMI/T4 may serve as a marker of adverse prognosis in breast cancer. However, the results of SMI/T11 in different cancer types were not consistent. A previous report detected that visceral fat density at T11 (not SMI/T11) could objectively predict the risk of hepatic decompensation and survival in patients with hepatocellular carcinoma undergoing transarterial chemoembolization (38). In contrast, one study found that patients with progressive disease had significantly lower SMI/T11 levels compared with stable disease in lung cancer (39). Similarly, another study reported that muscle mass at T8 level (not T10 level) was the best predictor of survival, and muscle mass at T12 showed no association with survival in lung cancer (40). In line with prior studies, our results revealed that PMI/T4 may be more helpful in survival prediction than SMI/T11 in patients with breast cancer. The muscle index on the different thoracic vertebral levels exhibited different predictive capacities for survival, due in part to the fact that pectoralis muscles at T4 support upper extremity and respiratory system function (40).

However, there are some limitations in this study. First, the patients in our study came from a single center, which limits the generalizability of the results. Second, similar to other retrospective findings, we were unable to determine the causal link between parameters. Third, the cut-off value of the PMI at the level of T4 was not a confirmed optimal cut-off value. In addition, measurement of muscle function was not included in our study. Finally, a few patients developed metastatic disease, thus partly limiting the analysis and conclusion. Further studies are needed.

In conclusion, PMI at the T4 level may serve as a marker of adverse prognosis in breast cancer.

## Data Availability Statement

The raw data supporting the conclusions of this article will be made available by the authors, without undue reservation.

## Author Contributions

W-jH, WW, and M-lZ worked together to conceive and design the experiment. Q-Cj, XZ, SF, Y-xL, and J-rY completed the data collection together. S-dM and M-lZ completed the Data analysis together. W-jH wrote and edited the manuscript alone. R-tW and W-jH responsible for the quality control of the study and the review of the manuscript. All authors read and approved the final manuscript.

## Funding

This work was supported by grants from the National Cancer Center climbing Foundation (NCC201908B09).

## Conflict of Interest

The authors declare that the research was conducted in the absence of any commercial or financial relationships that could be construed as a potential conflict of interest.

## Publisher’s Note

All claims expressed in this article are solely those of the authors and do not necessarily represent those of their affiliated organizations, or those of the publisher, the editors and the reviewers. Any product that may be evaluated in this article, or claim that may be made by its manufacturer, is not guaranteed or endorsed by the publisher.
